# Decreased serum levels of IL-27and IL-35 in patients with Graves disease

**DOI:** 10.20945/2359-3997000000227

**Published:** 2020-03-30

**Authors:** Malek-Hosseini Saeed, Kalantar Kurosh, Amirghofran Zahra, Dabbaghmanesh Mohamad Hossein, Rostamzadeh Davood, Mohammad Reza Ataollahi

**Affiliations:** 1 Department of Immunology School of Medicine Shiraz University of Medical Sciences Shiraz Iran Department of Immunology, School of Medicine, Shiraz University of Medical Sciences, Shiraz, Iran; 2 Autoimmune Diseases Research Center Shiraz University of Medical Sciences Shiraz Iran Autoimmune Diseases Research Center, Shiraz University of Medical Sciences, Shiraz, Iran; 3 Endocrine and Metabolism Research Center Nemazee Hospital Shiraz University of Medical Sciences Shiraz Iran Endocrine and Metabolism Research Center, Nemazee Hospital, Shiraz University of Medical Sciences, Shiraz, Iran; 4 Department of Immunology School of Medicine Fasa University of Medical Sciences Fasa Iran Department of Immunology, School of Medicine, Fasa University of Medical Sciences, Fasa, Iran

**Keywords:** Graves’ disease, IL-27, IL-35, anti-TPO, anti-Tg

## Abstract

**Objectives:**

Graves’ disease (GD) is an autoimmune disease causing the overproduction of the thyroid hormone from thyroid gland. This disease is mainly the result of the production of antibodies against TSH receptors. Cytokines play an important role in orchestrating the pathophysiology in autoimmune thyroid disease. The regulatory role of IL-12 on TH1 cells has been proven. IL-27 and IL-35, members of IL-12 cytokine family, are two cytokines that have been newly discovered. IL-35 has been identified as a novel immunosuppressive and anti-inflammatory cytokine while IL-27 has both inflammatory and anti-inflammatory functions. The objective of the current study was to examine the changes in the serum level of the foregoing cytokines in GD patients in comparison to healthy controls.

**Materials and methods:**

In this study, serum levels of IL-27 and IL-35 were determined by an ELISA method; anti TPO and anti Tg were measured by an RIA method in 40 new cases of Graves’s disease. The findings were compared with 40 healthy controls.

**Results:**

The results showed a significant difference between IL-27 and IL-35 regarding their serum levels with P values of 0.0001 and 0.024, respectively; anti TPO and anti Tg levels of the cases were also significantly different from controls (p < 0.001).

**Conclusion:**

The reduction in the serum levels of IL-27 and IL-35 in GD patients compared to normal subjects suggests the possible anti-inflammatory role of these cytokines in GD.

## INTRODUCTION

Grave’s disease (GD) is an autoimmune thyroid disease characterized by thyrotoxicosis, diffuse goiter and the presence of autoantibody against thyroid-stimulating hormone (TSH) receptor (1). The three major clinical signs of this disease are hyperthyroidism, ophthalmopathy and localized skin manifestations (2). GD is reported in 0.5% of the world population and its incidence is 5-10 times higher in women than in men (3). Although GD is a multifactorial disease whose etiology has not been fully fathomed, evidence shows that the imbalance between pro- and anti-inflammatory cytokines and production of aberrant autoantibodies play a pivotal role in the disease pathogenesis (1). Many studies have shown that pro-inflammatory cytokines such as interleukin (IL)-2, IL-8, IL-6, tumor necrosis factor (TNF)-α and IL-17 are increased in the serum of GD patients (4). The evaluation of the T helper (TH) subsets involvement in GD showed a TH2 bias immunological imbalance and an increase in TH1- and TH17-related cytokines compared to healthy controls (5-7).

Cytokines play a crucial role in triggering and coordinating inflammatory immune responses. Improper cytokine expression appears to influence the pathogenesis of many human diseases including thyroid autoimmune diseases. The immune regulation of two cytokines related to IL-12 family, namely IL-27 and IL-35, has been demonstrated in several autoimmune diseases (8). IL-27 is a heterodimeric cytokine comprised of Epstein-Barr virus (EBV)-induced gene 3 (EBI3) subunit (a protein linked to IL-12-p40) and the p28 subunit (9). Antigen presenting cells, including monocytes, macrophages and dendritic cells are the most important sources of IL-27 (10) that has both pro-inflammatory and anti-inflammatory activities (11). This cytokine is able to induce the proliferation of naive T cells and promote TH1 immune responses (12). Additionally, IL-27 regulates the production of anti-inflammatory cytokines such as IL-4, IL-10, and transforming growth factor beta (TGF-β) (13). IL-35 is a novel cytokine also belonging to IL-12 family. It is produced by regulatory T cells and plays an important role in suppressing the immune system (14). Regulation of cytokine network is a main target of various investigations in autoimmune inflammatory diseases (15,16). The immunoregulatory role of these two cytokines in GD is yet to be elucidated. Therefore, the aim of the present study was to generate a new insight on the role of these cytokines in GD development or pathogenesis. For this purpose, the levels of IL-27 and IL-35 cytokines were compared between GD patients and healthy controls. The possible relationship between these cytokines and the presence of anti-thyroid antibodies including anti-thyroid peroxidase (anti-TPO) and anti-thyroglobulin (anti-Tg) antibodies was further assessed in GD patients.

## MATERIALS AND METHODS

### Study population

Forty new cases of GD referring to the Motahari outpatient clinics, Shiraz University of Medical Sciences, participated in this study. Patients were diagnosed by an endocrinologist on the basis of clinical manifestations, biochemical criteria of thyrotoxicosis (TSH < 0.05 mIU/L and increased free T3 and/or free T4 levels) and the presence of TSH receptor antibodies (TRAb). The Graves orbitopathy (GO) was checked in GD patients according to the NO SPECS grading system. Moreover, 40 healthy subjects matched in age and sex with the patient group were included in the study. The controls did not have any history of GD or other autoimmune diseases. Written informed consent was obtained from all the subjects, and the study was reviewed and approved by the Ethics Committee of Fasa University of Medical Sciences.

### Sample collection

From all study participants, 5ml peripheral blood sample was collected. Following centrifugation at 3000 rpm for 10 min, the sera were separated, aliquoted and kept at -70 ºC until further use.

### Measurement of anti-Tg and anti-TPO and TSH receptor antibodies

Serum levels of anti-Tg and anti-TPO antibodies were evaluated by radioimmunoassay (RIA) using anti-hTg [^125^I] RIA kit (RK-8CT) and anti-hTPO [^125^I] RIA kit (RK-36CT) obtained from Institute of ISOTOPE, Hungary. RIA protocol was carried out according to the manufacturer’s instruction. Briefly, samples and calibrators were incubated together with biotin labelled anti-Tg (or biotin labelled anti-TPO for anti-TPO assay) and ^125^ITg (or ^125^I TPO) in the streptavidin-coated tubes. Following incubation, the content of the tubes was aspirated and the bound activity was measured in a gamma counter. The concentrations of anti-Tg and anti-TPO antibodies were inversely proportional to the radioactivity measured in the test tubes. The concentration was read off the calibration curve generated through plotting the binding values against a series of calibrators containing known amounts of anti-Tg or anti-TPO.

Serum levels of TSH receptor antibody (TRAb) were evaluated by competitive ELISA technique (Medizym^®^ TRAb clone from Medipan, Dahlewitz/Berlin, Germany) according to the manufacturer’s instruction. Briefly, samples, control and calibrators were added to respective tubes and incubated for 120 min at room temperature. After incubation, wells were aspirated and washed to remove any residual droplets. Next, monoclonal human antibody biotin complex (M22) was added. The incubation and washing steps was repeated. Conjugate solution was then added to the test tubes. After that, the incubation and washing steps were performed, and the substrate solutions were then added to each well. Finally, the optical density was read at 450 nm. The concentrations of TRAb were inversely proportional to the enzymatic activity measured in the test tubes.

### Measurement of serum IL-27 and IL-35 levels

IL-27 and IL-35 cytokine serum levels were measured using ELISA kits from R&D Systems, USA (for IL-27) and Zell Bio, Germany (for IL-35) according to the manufacturer’s instructions. The sensitivity of tests was 12.8 pg/mL (IL-27) and 0.015 ng/mL (IL-35). Brieﬂy, for IL-27 measurement, 100 μL of capture antibody was coated in each well of a 96-well microplate overnight at room temperature (RT). After washing and blocking, 100 μL serum samples of patients and controls were added for 2 h at RT. Plates were washed again and 100 μL of the detection antibody and then 100 μL of the working dilution of streptavidin-horseradish peroxidase (HRP) were added for 20 min. The substrate solution (100 μL) was then added for 20 min, and finally the reaction was stopped by adding a stop solution to each well. Regarding IL-35 serum levels, 40 μL samples along with 10 μL anti-IL-35 biotinylated antibody and 50 μL streptavidin-HRP were simultaneously added to a 96-well microplate pre-coated with anti-IL-35 antibody. After 60 min at 37 ºC and then washing, 100 μL of substrate solution was added for 10 min at 37 ºC, and the reaction was then stopped via a stopping solution. Ultimately, for both cytokines, the optical density of samples was read by use of a microplate reader at 450 nm. The levels of cytokines were extrapolated from the related standard curve.

### Statistical analysis

Statistical analysis and plotting of the graphs were performed using SPSS version 23 (SPSS Inc. Chicago, USA) and GraphPad Prism (GraphPad software Inc. CA) software, respectively. The non-parametric Mann-Whitney U-test was used to compare the serum levels of cytokines between patients and controls. The correlation between the serum levels of IL-27 and IL-35 and autoantibodies was examined using the Pearson test. P-values less than 0.05 were considered significant.

## RESULTS

### Patient’s demographics

In this study, 31 patients (77.5 %) were females and 9 were males. The mean age of patients was 35.9 years (range: 20-60 years). Of the 40 healthy controls, 30 (75%) were females and 10 (25%) were males. The mean age and age range of controls were 36.5 and 20-60 years, respectively.

### Detection of anti-TPO and anti-Tg antibodies and thyroid hormones

**Figure 1:**
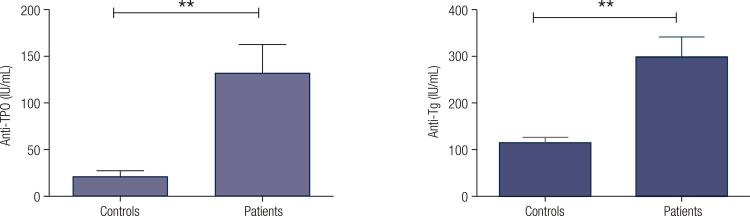
Anti-TPO and Anti-Tg levels in GD patients and healthy group. Serum antibody levels in GD patients (n = 40) and control group (n = 40) were measured by radioimmunoassay. Anti-TPO and anti-Tg levels were significantly higher in GD patients (** p < 0.001).

In GD patients, the levels of free T4, free T3, TSH and TRAb were 32.1 ± 9.30 pmol/l, 12.5 ± 6.23 pmol/l, 0.1 ± 0.9 mIU/mL and 5.1 ± 0.2 mIU/mL, respectively. Twenty-two patients (55%) were positive for anti-TPO antibody and 33 (82.5%) were positive for anti-Tg antibody. All normal subjects were negative for these two antibodies. All GD patients were positive for TRAb (Table 1). Higher levels of anti-TPO and anti-Tg antibodies were observed in patients compared to the controls (p < 0.001, Figure 1).

**Table 1:** Anti-Tg and anti-TPO antibody levels in patients and healthy controls

		Anti-Tg (IU/mL)		Anti-TPO (IU/mL)	
		
	Number	Range	Mean ± SE	Range	Mean ± SE
Grave’s patients	40	12.1-696	135 ± 30.5	39.9-1177	299 ± 42.4
Control group	40	7.3-59.1	22.4 ± 1.01	31.1-223	114.4 ± 12.2

### Serum levels of IL-27 and IL-35

**Figure 2:**
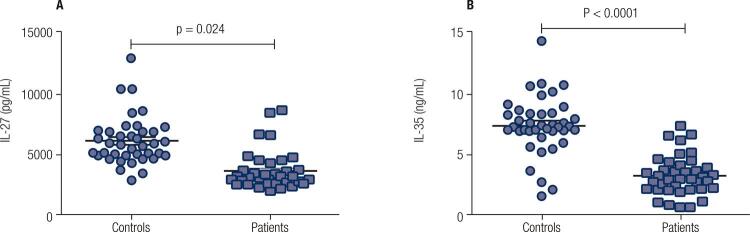
Comparison of the serum levels of IL-27 (p = 0.024) and IL-35 (p < 0.0001) in GD patients versus the controls. Cytokine levels in GD patients (n = 40) and control group (n = 40) were measured by ELISA. Compared to the normal group, the IL-27 and IL-35 were decreased in GD patients.

Analysis of the IL-27 cytokine level in the studied subjects showed a lower IL-27 serum concentration in the patients compared with the control group (3536 ± 246.3 *versus *6013 ± 314.3 pg/mL). As shown in Figure 2A, the difference between these levels was significant (p = 0.024). Similarly, a lower IL-35 level was observed in the patients (3.02 ± 0.25 ng/mL) in comparison to the controls (7.12 ± 0.36 ng/mL), (p < 0.0001) (Figure 2B). Statistical analysis showed no significant correlation between the IL-27 and IL-35 cytokines neither in patients nor in controls.

### Relationship between IL-27, IL-35, anti-thyroid antibodies, TRAb and the grade of patients

The relationship between the levels of these two cytokines and the concentration of anti-TPO, anti-Tg antibodies and TRAb levels was examined using the Pearson correlation test. In patients, age did not have any correlations with the levels of IL-27 and IL-35 and TRAb, anti-TPO, and anti-Tg antibodies (Table 2).

**Table 2:** Correlation between IL-27 and IL-35 cytokines and anti-thyroid antibodies and age in patients

	AGE	Anti-TPO	Anti-Tg	trab
IL-27	-0.232	0.084	0.178	-0.04
	0.667	0.608	0.297	0.806
IL-35	0.150	-0.047	-0.120	0.165
	0.345	0.772	0.456	0.308

**Figure 3:**
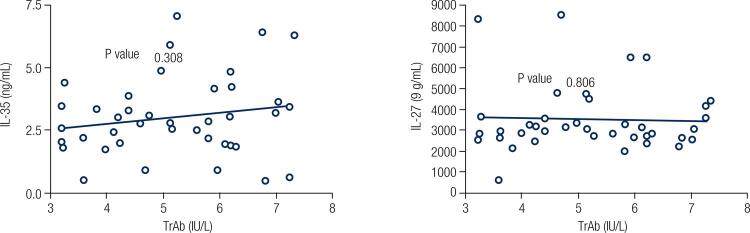
Correlation between IL-27 and IL-35 cytokine levels and level of TRAb in GD patients.

There was no significant correlation between any of the measured IL-35, IL-27, TRAb, anti-Tg and anti-TPO factors in the subjects and the disease grading parameter performed by the endocrinologist (Figure 3 and Table 3). No significant correlation was found between IL-27 and IL-35 serum levels and Graves orbitopathy (p = 0.74 and p = 0.2, respectively).

**Table 3:** Correlation between grade and IL-27, IL-35 cytokines, anti-thyroid antibodies and age in patients

		Anti TPO	Anti TG	age	IL35	IL27	TRAb
Grade	Pearson Correlation	-0.095	0.081	-0.263	-0.163	0.061	0.188
	Sig. (2-tailed)	0.631	0.681	0.175	0.416	0.788	0.337
	N	28	28	28	27	22	28

## DISCUSSION

GD is a TH2 predominant autoimmune illness defined by the presence of anti-TSH receptor antibody which motivates thyroid hormone secretion (17). The appearance of other antibodies against thyroid antigens such as anti-Tg and anti-TPO antibodies was reported in patients with hyperthyroidism (18). As it was observed in the present study, the serum levels of anti-Tg and anti-TPO antibodies in GD patients was significantly higher than those of control subjects, which is in line with previous reports (19).The generation of these antibodies can be attributed to the increased expression of the specific antigens and loss of tolerance in the organ (20).

Cytokines take part in the induction and effector steps of all inflammatory and immune responses, playing a critical role in the progression of autoimmune diseases. Excess, reduced, or improper cytokine responses significantly contribute to the formation of autoimmune inflammation (8). *In vitro* experiment analysis of intra-thyroidal lymphocytes including TSH-receptor specific T cells has revealed the predominance of the TH2 response in GD. In fact, following the initiation of TH2-related responses, the inflammatory process continues through Th1 cells. In this way, the cytokines such as IL-1a, IL-6 and TNF-α are generated by thyroid follicular cells as well as inflammatory cells. Moreover, increased gene expression of IL-l α, IL-I3, IL-6, TNF-α and IFN-γ was also shown in GD (5). On the other hand, IL-1β was able to induce the production of hyaluronan by thyroid epithelial cells and thyroid ﬁbroblasts, a process possibly contributing to the progression of goiter in GD (21). Cytokines of IL-12 family played important roles in the immune system and inflammation (22). IL-27 and IL-35 were relatively new members of the IL-12 family (23), and in the present study, the serum levels of these two cytokines in GD patients were reduced in comparison to normal individuals. IL-27 was shown to have two distinct inflammatory and anti-inflammatory functions (11). To our knowledge, no study has measured IL-27 in GD. Regarding other autoimmune diseases, IL-27 reduction has been reported in multiple sclerosis (MS) patients (24). After the interferon therapy of these patients with IL-27, increased serum levels of IL-27 were observed, and researchers predicted that with the administration of this cytokine, the recovery of MS disease would increase (24). Although increased levels of this cytokine were reported in patients with rheumatoid arthritis compared to healthy subjects (25), the role of IL-27 is complex and in addition to anti-inflammatory activity, its pro-inflammatory function should be considered (26). Among IL-27 pro-inflammatory functions, inhibition of Th17 cells and antagonistic effect on IL-6 activity were reported to be noteworthy (27,28). The dual function of IL-27 may be explained by the fact that this cytokine can be released from various cells such as antigen presenting cells under various conditions depending on the type of disease, cytokine network and the dominant cytokine profile. In this regard, IL-27 was proposed as a promising antiviral and anti inﬂammatory agent with apparent low toxicity risk as observed in animal and *in vitro* models (29).

In the present study, a significant reduction was observed in the IL-27 belonging to the sera of patients with GD in comparison to the healthy control group (P value = 0.024). The mean serum level of IL-27 in the patient group was approximately half of the control group. This result casts doubt on the inflammatory role of this cytokine in the development of GD while further confirming the anti-inflammatory properties of this cytokine as evidenced in previous studies on some autoimmune mice models such as uveoretinitis, multiple sclerosis and rheumatoid arthritis (30).

The IL-35 is secreted by activated antigen presenting cells including B cells, monocytes, macrophages and dendritic cells. This cytokine was initially introduced to be generated by Treg cells and play an essential role in the inhibitory function of these cells (31). Recently, the immunomodulatory role of IL-35 has been identified in certain inflammatory conditions (32). The main inhibitory effects of IL-35 were on TH1 and TH17 cells (31). Administration of IL-35 to mice with type 1 diabetes inhibited the disease (33). Moreover, the therapeutic effects of IL-35 on collagen-induced arthritis in the RA model were documented (34). In the only study on GD, the levels of IL-35 and TGF-β cytokine levels were reduced while IL-17A, IL-23, IL-6 serum levels were increased in Chinese GD patients compared with healthy controls (35), which is consistent with the present study . No correlations were observed between IL-35 and autoantibody levels in the patients of the current research. These results may suggest that this inhibitory cytokine is a therapeutic agent in GD. It is likely that the administration of IL-35 leads to the suppression of related inflammatory process in GD. This hypothesis should be verified in future studies on mice models of autoimmune thyroiditis.

## CONCLUSION

The reduction in the serum levels of IL-27 and IL-35 in GD patients compared to normal subjects suggests the possible anti-inflammatory role of these cytokines in GD, hence their use as therapeutic candidates for the treatment of GD patients in future.
